# Beneficial Effect of Insulin Treatment on Islet Transplantation Outcomes in Akita Mice

**DOI:** 10.1371/journal.pone.0095451

**Published:** 2014-04-17

**Authors:** Kazuhide Kikawa, Daisuke Sakano, Nobuaki Shiraki, Tomonori Tsuyama, Kazuhiko Kume, Fumio Endo, Shoen Kume

**Affiliations:** 1 Department of Pediatrics, Graduate School of Medical Sciences, Kumamoto University, Chuo-ku, Kumamoto, Japan; 2 Department of Stem Cell Biology, Institute of Molecular Embryology and Genetics (IMEG), Kumamoto University, Chuo-ku, Kumamoto, Japan; 3 Program for Leading Graduate Schools “HIGO (Health life science; Interdisciplinary and Glocal Oriented) Program,” Kumamoto University, Chuo-ku, Kumamoto, Japan; State University of Rio de Janeiro, Biomedical Center, Institute of Biology, Brazil

## Abstract

Islet transplantation is a promising potential therapy for patients with type 1 diabetes. The outcome of islet transplantation depends on the transplantation of a sufficient amount of β-cell mass. However, the initial loss of islets after transplantation is problematic. We hypothesized the hyperglycemic status of the recipient may negatively affect graft survival. Therefore, in the present study, we evaluated the effect of insulin treatment on islet transplantation involving a suboptimal amount of islets in Akita mice, which is a diabetes model mouse with an *Insulin 2* gene missense mutation. Fifty islets were transplanted under the left kidney capsule of the recipient mouse with or without insulin treatment. For insulin treatment, sustained-release insulin implants were implanted subcutaneously into recipient mice 2 weeks before transplantation and maintained for 4 weeks. Islet transplantation without insulin treatment did not reverse hyperglycemia. In contrast, the group that received transplants in combination with insulin treatment exhibited improved fasting blood glucose levels until 18 weeks after transplantation, even after insulin treatment was discontinued. The group that underwent islet transplantation in combination with insulin treatment had better glucose tolerance than the group that did not undergo insulin treatment. Insulin treatment improved graft survival from the acute phase (i.e., 1 day after transplantation) to the chronic phase (i.e., 18 weeks after transplantation). Islet apoptosis increased with increasing glucose concentration in the medium or blood in both the *in vitro* culture and *in vivo* transplantation experiments. Expression profile analysis of grafts indicated that genes related to immune response, chemotaxis, and inflammatory response were specifically upregulated when islets were transplanted into mice with hyperglycemia compared to those with normoglycemia. Thus, the results demonstrate that insulin treatment protects islets from the initial rapid loss that is usually observed after transplantation and positively affects the outcome of islet transplantation in Akita mice.

## Introduction

Diabetes is currently a global health problem. The World Health Organization (WHO) reports that 347 million people have diabetes worldwide. Diabetes is caused by the autoimmune destruction of pancreatic β cells (i.e., type 1 diabetes) or the combination of insulin resistance of all body organs and insulin secretion deficiency (i.e., type 2 diabetes).

Islet transplantation is a promising therapy for severely insulin-dependent diabetes patients in whom the endogenous insulin secretion is insufficient. As sustained insulin independence was reported in type 1 diabetes patients in the Edmonton protocol in 2000 [1], the incidence of islet transplantation has rapidly increased. However, islet transplantation has not yet become a standard therapy for diabetes because of donor shortages and the necessity of lifelong immunosuppressant drug use. Another important issue is the initial loss of many islets immediately after transplantation as a result of graft inflammation, immunorejection, apoptosis, or necrosis [2]–[4].

Efforts have been made to improve graft survival [5]. Suppression of immunorejection is the most important factor for a successful transplantation. A new immunosuppression trial has reported the combination of co-stimulation blockage via the CD80–CD86 pathways and thymoglobulin T-cell depletion [6]. In addition, some strategies are being developed to suppress inflammation. For instance, heparin and insulin infusions have been shown to significantly prevent instant blood-mediated inflammatory response (IBMIR) [7], the combination of anti-tumor necrosis factor (TNF)-α and interleukin (IL)-1 receptor blockage [8], and the inhibition of interferon (IFN)-γ [9] or caspase [2], [10], all of which improve the efficacy of islet engraftment. Moreover, the use of glucagon-like peptide-1 (GLP-1) analog improves human islet survival in culture [11]. Various types of scaffolds such as extracellular matrix protein-coated scaffolds [12] and microporous polymer scaffolds, which allow vascular ingrowth and nutrient diffusion from the host tissue [13], improve islet transplantation outcomes.

On the other hand, to overcome donor shortages, regenerative therapies using embryonic stem cells (ES cells) or induced pluripotent stem cells (iPS cells) are strong candidates for the treatment of diabetes [14]–[16]. In this field, several studies have focused on improving the extent of differentiation and maturation of ES or iPS cell-derived β cells [17], [18]. However, considering the issues described above, the establishment of an efficient procedure for improving graft survival is also important.

Prolonged or repeated exposure to elevated glucose concentrations has deleterious effects on the expressions of genes related to insulin production, insulin content, glucose-stimulated insulin secretion (GSIS), and β-cell viability [19]–[22]. Therefore, we hypothesized that the hyperglycemic status of recipients themselves is an obstacle to graft survival and that insulin treatment to recipients improves transplantation outcomes.

Accordingly, this study evaluated the effects of insulin treatment on the outcome of islet transplantation in Akita mice [23], which carry an *Insulin 2* (*Ins 2*) gene missense mutation [24] and exhibit insulin deficiency, thus making them an excellent model for diabetes with long-term sustained hyperglycemia.

## Materials and Methods

### Animals

This animal work is approved by the Institutional Review Board for Animal Care and Use of Kumamoto University. All animal procedures were conducted according to Kumamoto University guideline. Mice had access to food and water *ad libitum* except during fasting. Heterozygous Akita mice (C57BL/6J- *Ins2* ^WT/C96Y^ *Rag1* ^+/+^ or *Rag1* ^−/−^, male) were purchased from The Jackson Laboratory (Bar Harbor, ME, USA). Immunodeficient *Ins2* ^WT/C96Y^ *Rag1* ^−/−^ Akita mice were used only to evaluate GSIS. Polymerase chain reaction (PCR) was performed to genotype the *Ins 2* and *Rag1* genes, as described by the Jackson Laboratory. The fasting blood glucose levels and body weight were measured from 5 weeks of age in mice that had been fasted for 2 hours using a portable glucose monitor (Life check sensor; Gunze, Tokyo, Japan) by making an incision in the tail.

### Insulin Therapy

Insulin treatment was performed by implanting Linβit, sustained-release insulin implants (approximately 0.1 U d^−1^ implant^−1^; Linshin Canada Inc., Scarborough, Canada) [25]. These insulin implants are composed of an admixture of insulin and microrecrystallized palmitic acid, allowing sustained insulin release, which yields more consistent blood glucose levels than that obtained by daily insulin injections.

After brief isoflurane (2-chloro-2-(difluoromethoxy)-1,1,1-trifluoro-ethane) (Merck Animal Health, NJ, USA) anesthesia was administered, insulin implants were inserted subcutaneously in the nape of the neck. Mice weighing <20 g and 20–25 g received 2 and 3 implants, respectively. In the insulin-treated group, insulin implants were inserted between 8 and 10 weeks of age. Two weeks after implantation (i.e., 10–12 weeks of age), islet transplantations were performed. Insulin implants were removed 2 weeks after islet transplantation.

### Islet Isolation and Transplantation

Wild-type (WT) C57BL/6 mice were used as donors for islet transplantation, and heterozygous male Akita mice were used as recipients. Islets were isolated by the collagenase digestion of the pancreas as described previously [26] with some modifications. Briefly, donor mice were euthanized, and the pancreas was exposed by laparotomy. The common bile duct was ligated at the ampulla of Vater and cannulated using a needle (Natsume Seisakusho, Tokyo, Japan). Thereafter, 4–5 mL solution containing 2 mg/mL collagenase IV (GIBCO, CA, USA) was injected into the pancreas. The pancreas was subsequently removed, and the islets were collected using density gradient reagent Histopaque 1077 (Sigma-Aldrich, St. Louis, MO, USA), and handpicked under a stereomicroscope 2 or 3 times until a population of pure islets was obtained. Approximately 100 islets were obtained per mouse. Under isoflurane anesthesia, the lumbar region of the recipients was incised. Either 50 or 200 islets were transplanted under the left kidney capsule of the recipient using a 24-gauge catheter (NIPRO, Osaka, Japan) on the day of isolation. The lumbar incision was sutured after transplantation. Glucose monitoring was continued until 18 weeks after islet transplantation, when the grafts were removed.

### Islet Culture

Islets from WT C57BL/6 mice were used for islet culture. Isolated islets were handpicked and cultured in Dulbecco’s modified Eagle medium with various glucose concentrations supplemented with 10% fetal bovine serum, 100 µM nonessential amid acids, 2 mM l-glutamine, 50 units/mL penicillin, 50 µg/mL streptomycin, and 100 µM 2-mercaptoethanol. Islets were cultured for 24 hours on 24-well plates (Sumitomo Bakelite Co. Ltd., Tokyo, Japan).

### Intraperitoneal Glucose Tolerance Test (IPGTT) and GSIS Assay

Mice that had been fasted for 6 hours were used. Blood glucose levels were measured before (0 min) and 15, 30, 60, 90, and 120 min after intraperitoneal administration of 25% glucose solution (Wako, Osaka, Japan) at 2 g/kg body weight. Serum insulin levels were measured using a mouse insulin kit (Shibayagi, Gunma, Japan) before (0 min) and 30 min after glucose challenge.

### Insulin Tolerance Test (ITT)

Mice that had been fasted for 6 hours were used. Blood glucose levels were measured before (0 min) and 30 min after intraperitoneal administration of Humulin R (Eli Lilly, IN, USA) at 0.5 mU/g body weight. The relative rate of glucose decrease as a result of insulin treatment from each basic glucose level was calculated.

### Antibodies

The primary antibodies used were rat anti-insulin (R&D, Minneapolis, MN, USA), guinea pig anti-glucagon (Progen Biotechnik, Heidelberg, Germany), and rabbit anti-cleaved caspase-3 (Cell Signaling, MA, USA). Alexa 488 and Alexa 568 (Invitrogen, Paisley, UK) conjugated secondary antibodies were also used.

### Apoptosis Assay

For the apoptosis assay, caspase-3/7 activity was measured using the CellEvent Caspase-3/7 Green Detection Reagent (Invitrogen Life Technologies Co., Carlsbad, CA, USA) according to the manufacturer’s protocol. Apoptosis was also detected by the terminal deoxynucleotidyl transferase-mediated d-UTP nick-end labeling (TUNEL) method using an *in situ* cell death detection kit (Roche, Penzberg, Germany).

### Microarray Analysis

A MOE430 2.0 series probe array (GeneChip, Affymetrix, CA, USA) was used for microarray analysis. The expression analysis was performed using the Subio Platform (Subio, Tokyo, Japan).

### Microarray Data Accession Number

Microarray data are deposited under Gene Expression Omnibus (GEO) accession number GSE55764.

### Statistics

The results are presented as mean ± standard deviation (SD). Statistical significance was assessed by Student’s *t*-test. In addition, ANOVA was used to evaluate the differences in glucose levels among groups.

## Results

### Insulin Treatment Improved Islet Transplantation Outcomes

**Figure 1:**
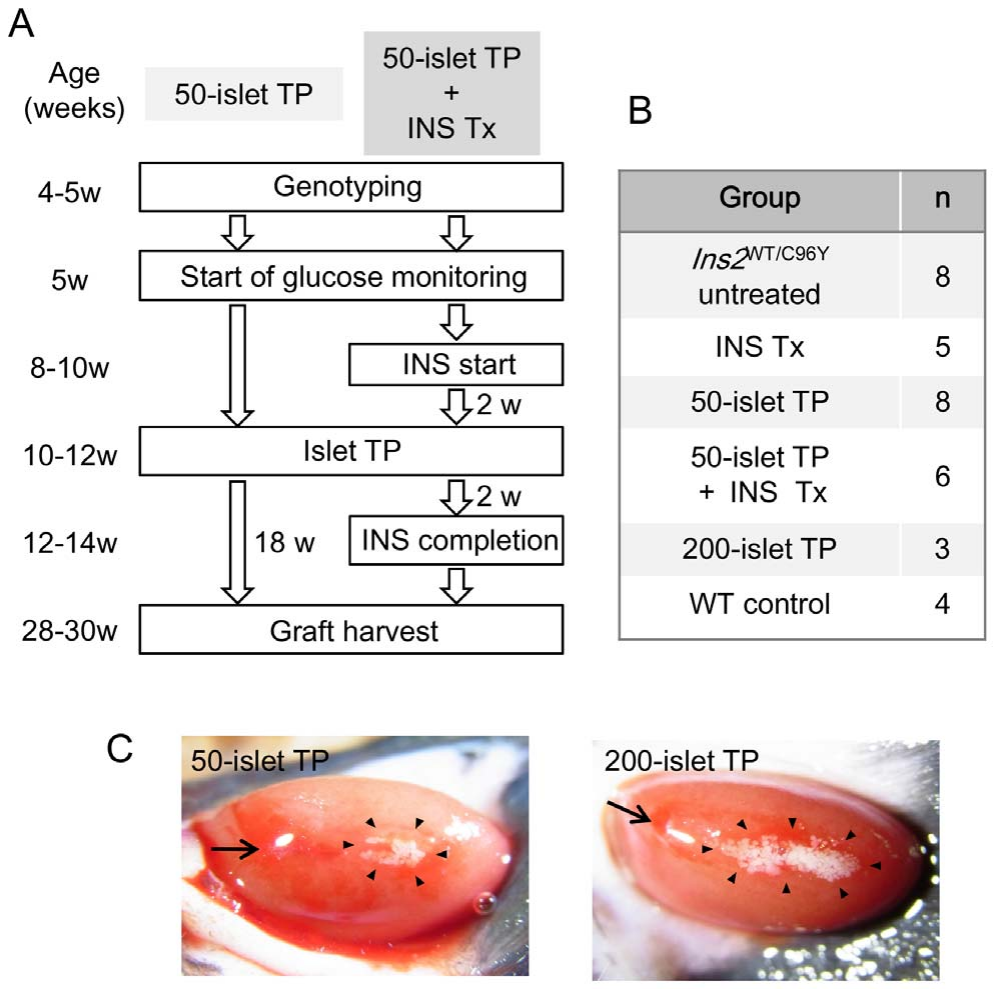
Groups and protocol. (A) Schematic drawing of the experiment for evaluating the effect of insulin treatment on islet transplantation. Heterozygous male Akita mice (*Ins2* ^WT/C96Y^) were used as recipients. Small insulin implants were used for insulin treatment. Glucose monitoring was started from 5 weeks of age. In mice transplanted with 50 islets without insulin treatment (50-islet TP), islet transplantation was performed at 10–12 weeks of age. In mice transplanted with 50 islets in combination with insulin treatment (50-islet TP+INS Tx), insulin implants were inserted when the mice reached 8–10 weeks of age. After 2 weeks, islet transplantation was performed (10–12 weeks of age). Implants were removed 2 weeks after islet transplantation (12–14 weeks of age). Glucose monitoring was continued until 18 weeks after islet transplantation (28–30 weeks of age). (B) Numbers of mice in each group. (C) Images of 50 (left) or 200 (right) islets transplanted under the left kidney capsule of Akita mice. Images were obtained immediately after transplantation. Arrows indicate catheter insertion sites, and arrowheads indicate the outer boundary of the transplanted islets.

Heterozygous Akita mice confirmed by genotyping were used in this experiment (Figure S1A in File S1). The protocol and numbers of mice in each group are summarized in Figure 1A and B. When islets were transplanted under the kidney capsule of the recipient (Figure 1C; arrows indicate the catheter insertion site), they were distributed along the direction of catheter insertion (Figure 1C; arrowheads indicate the outer boundary of the transplanted islets).

**Figure 2:**
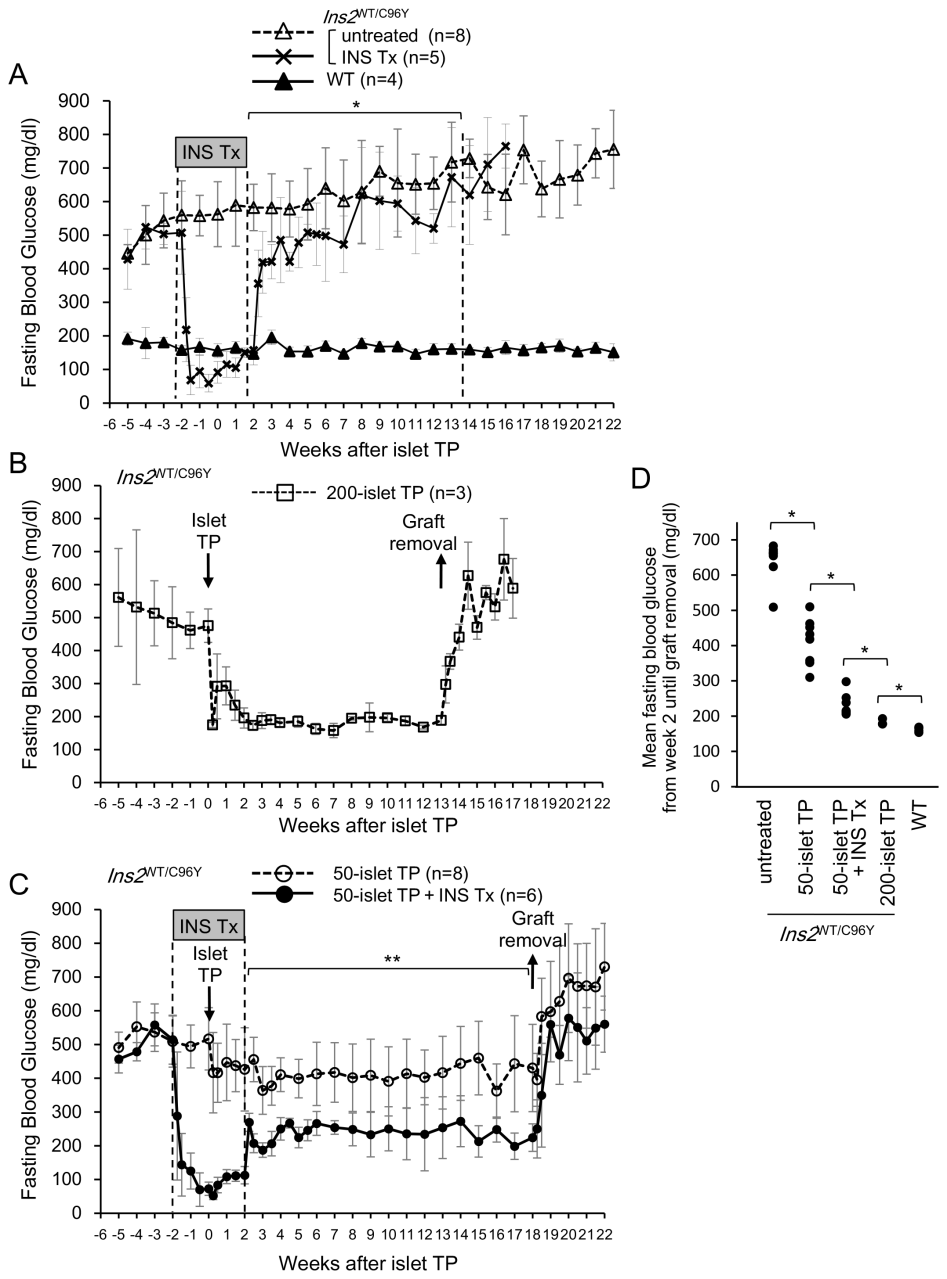
Insulin treatment improved islet transplantation outcomes. (A–C) Fasting blood glucose levels of Akita (*Ins2* ^WT/C96Y^, untreated) mice (broken line with open triangles), the INS Tx group (solid line with crosses), and C57BL/6 WT mice (solid line with closed triangles). (A) Insulin implants were inserted at week −2 and removed at week 2. There were significant differences between the INS-treated and INS-untreated groups from 2 to 14 weeks (*p<0.05, ANOVA). (B) Fasting blood glucose levels in the 200-islet TP group (broken line with open squares). Two hundred islets were transplanted at week 0, and removed at week 13. (C) Fasting blood glucose levels of the 50-islet group (broken line with open circles) and 50-islet TP+INS Tx group (solid line with closed circles). Insulin treatment significantly lowered the blood glucose levels (**p<0.01, ANOVA). (D) Average fasting blood glucose levels of individual mice in each group from the time of insulin implant removal (week 2) until graft removal. Each dot represents the mean values in 1 mouse. The 50-islet TP+INS Tx group had significantly lower average fasting blood glucose levels than the 50-islet TP group (*p<0.05, two-tailed unpaired Student’s *t-*test).

First, we tested the effects of insulin treatment on Akita mice. All male heterozygous Akita mice that did not receive any treatment gradually developed hyperglycemia spontaneously, as reported previously [27]–[29], and had fasting blood glucose levels reaching approximately 700 mg/dL at ∼20 weeks of age (i.e., week 10 in Figure 2A). In contrast, the fasting blood glucose levels of control WT C57BL/6 mice remained stable at approximately 150 mg/dL. Insulin treatment in Akita mice via insulin implants effectively stabilized blood glucose levels for 4 weeks to levels that were even lower than those in the WT control mice. The recurrence of hyperglycemia was observed upon removal of the insulin implants in these mice (Figure 2A). There were significant differences in fasting blood glucose levels between the groups with and without insulin implants from weeks 2–14 (*p<0.05, ANOVA). There were no differences in the IPGTT (Figure S2A, S2B in File S1) and ITT (Figure S2D in File S1) results 1 week after insulin implant removal between the groups treated with and without insulin implants. The insulin implants contained insulin from different species, which induced the production of anti-insulin antibodies in the recipients and interfered with the measurement of serum insulin ELISA in mice. Therefore, we used immunodeficient *Rag1* ^−/−^ Akita mice to avoid this interference and examined GSIS. The *Rag1* gene was confirmed by genotyping (Figure S1B in File S1). There was no increase of endogenous insulin secretion 1 week after discontinuing insulin treatment (Figure S2C in File S1). These results collectively indicate that insulin treatment did not affect the glucose tolerance of the endogenous islets and that the subjects were not insulin resistant.

Next, we confirmed the effects of transplanting 200 islets, which is the optimal number of islets for reversing hyperglycemia, in the absence of insulin treatment. Transplanting 200 islets improved fasting blood glucose levels in all recipient mice, and normoglycemia was maintained from week 2 up to week 13 after transplantation. Upon graft removal, hyperglycemia recurred (Figure 2B).

We subsequently tested the hypothesis that insulin treatment improves the transplantation outcomes with a suboptimal number of islets. Mice that were transplanted with 50 islets alone exhibited average fasting blood glucose levels exceeding 300 mg/dL. In contrast, mice transplanted with 50 islets in combination with insulin treatment exhibited significantly improved fasting blood glucose levels. Even after discontinuing insulin treatment, fasting blood glucose levels in mice remained below 300 mg/dL until 18 weeks post-transplantation (Figure 2C). Only 1 mouse exhibited temporal hyperglycemia (>300 mg/dL) from weeks 9 to 15, but blood glucose levels remained <300 mg/dL thereafter. There were significant differences in fasting blood glucose levels between the groups treated with and without insulin implants from weeks 2 to 18 (*p<0.01, ANOVA). These data strongly suggest that insulin treatment improved islet transplantation outcomes. The mean fasting blood glucose level of each mouse from week 2 (corresponding to the time of insulin implant removal) until graft removal was determined. All mice transplanted with 50 islets in combination with insulin treatment exhibited significant improvements in the average blood glucose levels (<300 mg/dL) compared to those that did not receive insulin treatment (>300 mg/dL) (Figure 2D).

### Insulin Treatment Improved Glucose Tolerance after Islet Transplantation

**Figure 3:**
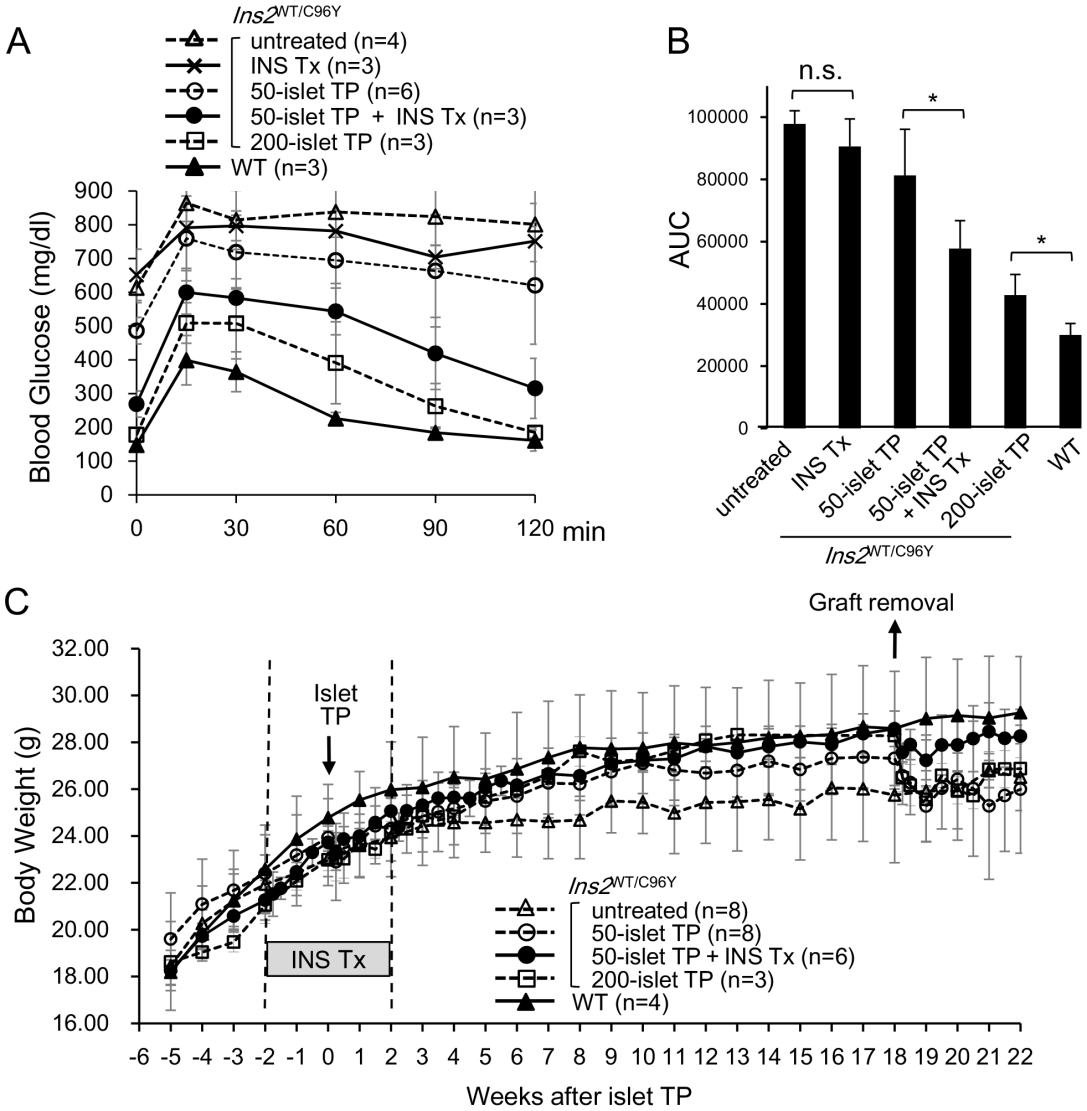
Glucose tolerance test of mice receiving 50-islet transplantation with insulin treatment showed improvment compared to those without insulin treatment. (A) An IPGTT was performed 9 weeks after transplantation. Blood glucose was measured 0, 30, 60, 90, and 120 min after glucose challenge. Blood glucose >900 mg/dL was recorded as “high” and plotted as 900 mg/dL. (B) Areas under the curve for each graph in (A). A significant difference was observed between mice that underwent 50-islet transplantation with and without insulin treatment (*p<0.05, two-tailed unpaired Student’s *t-*test). n. s., not significant. (C) Changes in the average body weight of each group.

The IPGTT was performed to evaluate glucose tolerance 9 weeks after islet transplantation. In the group that received 200 islets, the average blood glucose level at 120 minutes was similar to that seen in the WT control group. In the group that received 50 islets without insulin treatment, the average blood glucose level remained high and only decreased slightly 120 minutes after glucose challenge. However, in mice receiving both 50 islets and insulin treatment, the average blood glucose levels decreased significantly during the course of 120 minutes after glucose challenge, but were nevertheless higher than those of the WT control and 200-islet transplantation groups. The average blood glucose levels of the insulin-treated group (i.e., without islet transplantation) did not differ from those of the untreated group, again suggesting that insulin treatment alone did not affect glucose metabolism in the recipient mice (Figure 3A). The area under the curve (AUC) of the IPGTT results shows that glucose tolerance in mice transplanted with 50 islets in combination with insulin treatment improved significantly compared to mice that did not undergo insulin treatment (Figure 3B).

Thereafter, we compared the average body weight between groups. Weight gain in the untreated group was low because of hyperglycemia. The average body weight of the WT group increased rapidly at first and continued to increase at a slower rate thereafter. After islet transplantation, weight gain in all transplantation groups improved significantly, and increased to a level similar to that observed in the WT group. However, body weight decreased after graft removal, concurrent with the recurrence of hyperglycemia (Figure 3C).

### Islet Apoptosis Increased in High-glucose Concentrations

**Figure 4:**
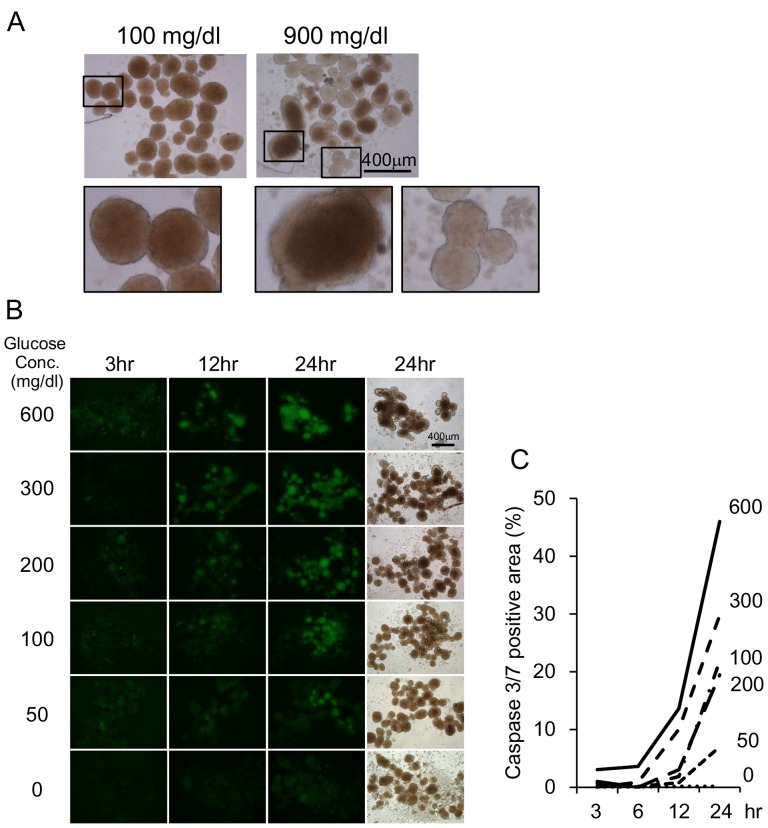
Islet apoptosis increased with increasing glucose concentration. (A) Islets were cultured in low-glucose (100 mg/dL, left) and high-glucose (900 mg/dL, right) media for 24 hours. Lower panels show high-magnification images. (B) The caspase-3/7–positive area increased after a 24-hour exposure to various glucose concentrations from 0 to 600 mg/dL. (C) Quantitative analysis of Figure 5B: media with glucose concentrations of 0 mg/dL (dotted line), 50 mg/dL (small broken line), 100 mg/dL (dot-broken line), 200 mg/dL (large broken line), 300 mg/dL (middle broken line), and 600 mg/dL (solid line) were used.

Next, we examined the mechanism underlying graft loss in hyperglycemic conditions. As some of the mice reached blood glucose concentrations of approximately 900 mg/dL (Figure 2A), we compared the effects of glucose by culturing the islets in low-glucose (100 mg/dL) and high-glucose (900 mg/dL) media for 24 hours. Islets cultured in low-glucose medium maintained their shape and color (Figure 4A, Left). However, necrosis was observed in the center of some islets, particularly the large islets, cultured in the high-glucose medium. Small islets were translucent and formed aggregates with each other (Figure 4A, right).

We subsequently determined whether apoptosis is involved in the morphological changes of islets under high-glucose conditions. Islets were cultured in media with various glucose concentrations. The caspase-3/7–positive area increased over time with increasing glucose concentrations. In contrast, the islets cultured in low-glucose conditions (i.e., 0 or 50 mg/dL) exhibited smaller apoptotic areas (Figure 4B, 4C).

### Graft Survival Improved as a Result of Insulin Treatment

**Figure 5:**
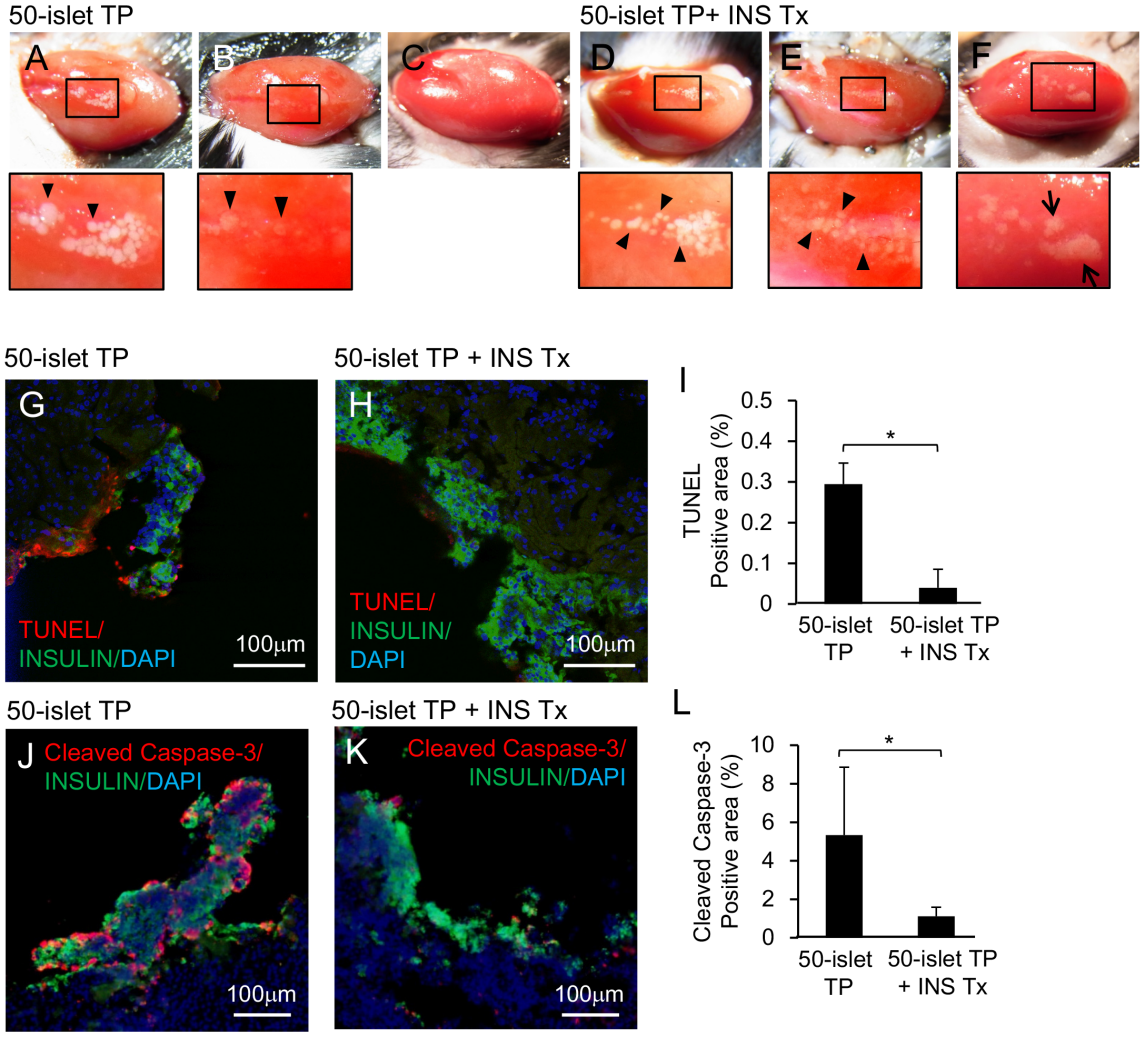
Graft survival improved as a result of insulin treatment. Kidneys from mice in the 50-islet TP group at (A) the time of transplantation, (B) day 1, and (C) week 18. (A) and (B) are from the same mouse. Kidneys from mice in the 50-islet TP+INS Tx group at (D) the time of transplantation, (E) day 1, and (F) week 18. (D) and (E) are from the same mouse. Lower panels show high-magnification images. Arrowheads indicate each islet, and arrows show aggregated islets. (G–L) Grafts were removed 6 hours post-transplantation and analyzed by TUNEL assay (G–I) or stained with anti-cleaved caspase-3 (J–L). Grafts of the 50-islet TP and 50-islet TP+INS Tx groups were compared (*p<0.05, two-tailed unpaired Student’s *t-*test). (I, L) Proportions of the number of positive cells to total cells in the grafts. DAPI (blue), insulin (green), and TUNEL or anti-cleaved caspase-3 (red).

Next, we investigated the grafts during the acute and chronic phases. In the 50-islet transplantation group (Figure 5A; immediately after transplantation under the kidney capsule), most islets disappeared on day 1 (Figure 5B) and it was almost impossible to identify the graft 18 weeks post-transplantation (Figure 5C). However, when 50-islet transplantation was combined with insulin treatment (Figure 5D, immediately after transplantation under the kidney capsule), approximately half of the transplanted islets remained detectable on day 1 (Figure 5E); at week 18, the grafts remained and appeared to form aggregates with each other (Figure 5F).

The graft area on day 1 was smaller in the mice that received 50 islets without insulin treatment than those with insulin treatment. Next, the proportions of cells expressing cleaved caspase-3 and TUNEL-positive cells were determined 6 hours post-transplantation. The proportions of cells positive for both apoptosis markers were higher in the group without insulin treatment (Figure 5G–L). Thus, these results suggest that insulin treatment improved graft survival.

To study the molecular mechanism underlying the improved islet survival, expression profile analyses was performed to analyze the differences between the grafts transplanted into hyperglycemic and normoglycemic mice at 6 hours post-transplantation. Genes with gene ontology (GO) terms classified as “immune response”, “chemotaxis,” and “inflammatory response” were significantly upregulated in grafts transplanted into hyperglycemic mice (Tables 1–4). These results suggest that hyperglycemia increased the inflammatory cytokine levels and immune responses *in vivo*.

**Table 1:** GO term analysis of genes upregulated >5-fold in hyperglycemic Akita mice compared to normoglycemic mice.

Gene Ontology Biological Process	P-value
Immune response	5.04E-09
Chemotaxis	1.32E-06
Inflammatory response	3.86E-06
Complement activation, classical pathway	1.88E-05
Cell adhesion	1.67E-04
Female pregnancy	1.17E-03
G-protein coupled receptor protein signaling pathway	1.49E-03
Innate immune response	1.61E-03
Integrin-mediated signaling pathway	6.75E-03
Cell proliferation	1.01E-02
Signal transduction	1.51E-02

**Table 2:** Upregulated genes (>5-fold) classified as ‘immune response genes’.

Gene Title	Gene Symbol	High/Normal
chemokine (C-X-C motif) ligand 13	Cxcl13	84.14
chemokine (C-C motif) ligand 24	Ccl24	24.85
chemokine (C-C motif) ligand 9	Ccl9	18.61
complement component 3a receptor 1	C3ar1	13.99
platelet factor 4	Pf4	10.29
chemokine (C-C motif) ligand 6	Ccl6	9.82
chemokine (C-C motif) ligand 7	Ccl7	9.28
chemokine (C-C motif) ligand 2	Ccl2	7.01
interleukin 16	Il16	6.87
complement component 3a receptor 1	C3ar1	6.83
kit oncogene	Kit	6.27
complement component 5a receptor 1	C5ar1	5.70
chemokine (C-C motif) ligand 12	Ccl12	5.61
Chemokine (C-C motif) receptor 9	Ccr9	5.35
chemokine (C-X-C motif) ligand 3	Cxcl3	5.28
formyl peptide receptor 2	Fpr2	5.24
chemokine (C-X-C motif) ligand 11	Cxcl11	5.22

**Table 3:** Upregulated genes (>5-fold) classified as ‘chemotaxis genes’.

Gene Title	Gene Symbol	High/Normal
chemokine (C-X-C motif) ligand 13	Cxcl13	84.14
galanin	Gal	48.58
chemokine (C-C motif) ligand 24	Ccl24	24.85
oxidized low density lipoprotein (lectin-like) receptor 1	Olr1	19.78
interleukin 17D	Il17d	18.52
CD44 antigen	Cd44	15.98
sphingosine-1-phosphate receptor 3	S1pr3	15.17
CD163 antigen	Cd163	14.65
selectin, platelet	Selp	13.98
histamine receptor H4	Hrh4	11.96
chemokine (C-C motif) receptor 1	Ccr1	9.64
thrombospondin 1	Thbs1	9.47
chemokine (C-C motif) ligand 7	Ccl7	9.28
C-type lectin domain family 7, member a	Clec7a	7.79
urocortin	Ucn	7.15
chemokine (C-C motif) ligand 2	Ccl2	7.01
interleukin 1 alpha	Il1a	6.77
lymphocyte antigen 86	Ly86	6.54
CD14 antigen	Cd14	6.45
toll-like receptor 13	Tlr13	6.38
thrombospondin 1	LOC640441///Thbs1	5.93
neutrophil cytosolic factor 1	Ncf1	5.85
chemokine (C-C motif) ligand 12	Ccl12	5.61
complement component 4B (Childo blood group)	C4b	5.45
chemokine (C-X-C motif) ligand 3	Cxcl3	5.28
chemokine (C-X-C motif) ligand 11	Cxcl11	5.22
chitinase 3-like 3///chitinase 3-like 4	Chi3l3///Chi3l4	5.07

**Table 4:** Upregulated genes (>5-fold) classified as ‘inflammatory response genes’.

Gene Title	Gene Symbol	High/Normal
chemokine (C-X-C motif) ligand 13	Cxcl13	84.14
CD79B antigen	Cd79b	35.78
mannose-binding lectin (protein C) 2	Mbl2	30.72
tumor necrosis factor receptor superfamily, member 13b	Tnfrsf13b	29.84
histocompatibility 2, blastocyst	H2-Bl	25.66
chemokine (C-C motif) ligand 24	Ccl24	24.85
CD55 antigen	Cd55	19.84
oxidized low density lipoprotein (lectin-like) receptor 1	Olr1	19.78
chemokine (C-C motif) ligand 9	Ccl9	18.61
C-type lectin domain family 4, member e	Clec4e	17.11
histocompatibility 2, class II, locus Mb2	H2-DMb2	16.52
interleukin 12a	Il12a	15.99
retinoic acid early transcript 1, alpha	Raet1a	14.72
proteoglycan 4 (megakaryocyte stimulating factor, articular superficial zone protein)	Prg4	12.19
complement component 1, q subcomponent, beta polypeptide	C1qb	11.22
interleukin 1 family, member 10	Il1f10	10.43
platelet factor 4	Pf4	10.29
C-type lectin domain family 4, member d	Clec4d	10.21
chemokine (C-C motif) ligand 6	Ccl6	9.82
chemokine (C-C motif) ligand 9	Ccl9	9.60
thrombospondin 1	Thbs1	9.47
chemokine (C-C motif) ligand 7	Ccl7	9.28
tumor necrosis factor (ligand) superfamily, member 14	Tnfsf14	9.15
C-type lectin domain family 7, member a	Clec7a	7.79
histocompatibility 2, M region locus 10.1	H2-M10.1	7.62
CD1d1 antigen	Cd1d1	7.41
complement component 1, q subcomponent, C chain	C1qc	7.40
Wiskott-Aldrich syndrome homolog (human)	Was	7.24
2′-5′ oligoadenylate synthetase 1E	Oas1e	7.19
linker for activation of T cells family, member 2	Lat2	7.19
complement factor properdin	Cfp	7.13
chemokine (C-C motif) ligand 2	Ccl2	7.01
interleukin 1 alpha	Il1a	6.77
lymphocyte antigen 86	Ly86	6.54
complement component 1, q subcomponent, alpha polypeptide	C1qa	6.53
tumor necrosis factor receptor superfamily, member 13c	Tnfrsf13c	6.52
zeta-chain (TCR) associated protein kinase	Zap70	6.48
CD14 antigen	Cd14	6.45
toll-like receptor 13	Tlr13	6.38
tumor necrosis factor (ligand) superfamily, member 9	Tnfsf9	6.16
histocompatibility 2, O region alpha locus	H2-Oa	6.10
Fas ligand (TNF superfamily, member 6)	Fasl	5.84
chemokine (C-C motif) ligand 12	Ccl12	5.61
complement component 4B (Childo blood group)	C4b	5.45
thrombospondin 1	Thbs1	5.43
chemokine (C-X-C motif) ligand 3	Cxcl3	5.28
chemokine (C-X-C motif) ligand 11	Cxcl11	5.22
complement receptor 2	Cr2	5.13

## Discussion

The results show that insulin treatment improved the islet transplantation outcomes. Significant improvements in blood glucose levels and glucose tolerance were observed by transplanting 50 islets, which is a suboptimal amount, combined with insulin treatment. Furthermore, insulin treatment increased graft survival in the most acute phase as well as the chronic phase. In *in vitro* islet cultures and *in vivo* grafts, islet apoptosis increased with increasing glucose concentration. Moreover, expression profile analyses suggested that hyperglycemic mice had disadvantages in terms of triggering more extensive inflammation and immune responses.

As streptozotocin (STZ, an *N*-nitroso derivative of glucosamine) administration damages β cells and induces hyperglycemia, it is widely used in experimental animal models of type 1 diabetes. The severe loss of β cells in mice treated with high doses of STZ can be lethal [30]. At low doses of STZ, β-cell loss is recovered by spontaneous β-cell regeneration and the hyperglycemia then improves [31]–[35]. The narrow dosage range for STZ to maintain hyperglycemia is a disadvantage for this model. The effects of insulin treatment in islet transplantation in studies using STZ diabetes models are inconsistent. Some reports show that insulin treatment improves the outcome of islet transplantation [36]–[39], β-cell survival [38], [39], and the glucose responsiveness of grafts [37]. However, others report that the beneficial effect of insulin treatment is lost within 4 weeks after treatment [40] and that rats with long-term (i.e., 6 months) diabetes, transplanted with islets in combination with insulin treatment, exhibit impaired glucose induced insulin secretion, thus indicating that insulin treatment has no beneficial effects [41]. Previous studies show that even with insulin treatment during islet transplantation, initial β-cell death is observed and β-cell masses are similar between islets transplanted into normoglycemic and hyperglycemic recipients. Thus, it was concluded that insulin treatment does not improve the initial preservation of transplanted β-cell mass in the initial days after transplantation [38], [39].

Accordingly, we hypothesized that the inconsistent results regarding insulin treatment are attributable to the instability of the STZ diabetes models. To overcome this, we used Akita mice, which are a non-obese, insulin-deficient diabetes model with no insulin resistance. The missense mutation (Cys^96^Tyr) in the *Ins 2* gene on chromosome 7 leads to pancreatic β-cell apoptosis [24]. Homozygous Akita mice exhibit sufficient levels of hyperglycemia and failure to thrive, and die within 3 months. Heterozygous Akita mice are reported to develop diabetes before 10 weeks of age. Diabetes symptoms progress in males, whereas females exhibit only mild symptoms. On histological examination, the pancreas of diabetic mice is extremely atrophic and largely devoid of granulated β cells in the absence of autoimmune insulitis. Therefore, Akita mice are excellent model subjects for islet transplantation studies [29]. We confirmed that heterozygous male Akita mice consistently exhibited hyperglycemia starting from 5 weeks of age (corresponding to week −5 in Figure 2A).

The present study used insulin implants that provide sustained insulin release and achieve more stable blood glucose levels as compared to daily insulin injections. The results show that in the absence of insulin treatment, the transplantation of 50 islets was insufficient and that 200 islets could achieve normoglycemia. This finding is concordant with a previous study on STZ-diabetic C57BL/6 mice [36].

There are several possible mechanisms underlying graft loss in hyperglycemic conditions. During isolation and following transplantation, islets experience ischemia due to the loss of oxygen supply from blood vessels [42], [43]. Furthermore, hyperglycemia is reported to increase oxygen consumption [44], [45]. Therefore, hyperglycemia exacerbates hypoxia and may cause both necrosis and apoptosis. Moreover, a normalized blood glucose level may suppress oxygen consumption. A caspase inhibitor is reported to improve graft survival after islet transplantation in mice [2], [10], supporting our hypothesis that apoptosis is involved in graft loss. The present study shows that islet apoptosis increased with increasing glucose concentration *in vitro*. Although apoptosis also occurred to some extent even at normal blood glucose levels, the results show that lower glucose concentrations triggered lower levels of apoptosis. In addition, the use of insulin implants maintained the blood glucose levels of Akita mice at a lower level than the WT mice, which might have positively affected graft survivals. Moreover, the expression profile analysis of grafts revealed that genes related to immune response, chemotaxis, and inflammatory response were specifically upregulated when islets were transplanted into mice with hyperglycemia, but not observed when transplanted into mice with normoglycemia. Inflammatory cytokine concentrations increased as a result of hyperglycemia in humans [46]. Furthermore, it was reported that β cells themselves produce cytokines that recruit proinflammatory monocytes/macrophages to the islets [47] or lead to IBMIR [48]. Compared to islets *in vitro*, islets transplanted *in vivo* appear to be damaged to a greater extent, resulting in a significant reduction in islet numbers. The present results suggest that islets transplanted *in vivo* are exposed to both inflammatory and immune responses, and that lowering of the blood glucose levels might suppress inflammatory reactions and protect against cell death.

Recent advances enable the induction of stem cells, such as ES and iPS cells, to differentiate into insulin-producing cells, which may serve as an alternative cell source for cell transplantation therapy in diabetes. However, there is a lack of improvement in graft survival rate as well as the maturity of ES/iPS cell-derived β cells. Thus, the protocol reported herein has implications for future studies of diabetes treatments including those using stem cells.

In conclusion, insulin treatment positively affects the outcome of islet transplantation in Akita mice, without influence the endogenous islets. Insulin treatment protects engrafted islets from the initial rapid loss after transplantation. Islet apoptosis increases with increasing glucose concentrations both *in vitro* and *in vivo*. The protective effect of normoglycemia in *in vivo* conditions can be explained by decreased inflammatory and immune responses.

## Supporting Information

File S1 **File S1 includes the following: Figure S1. Genotyping of Akita mice.** (A) PCR analysis of the *Ins 2* gene. The PCR product of the WT gene was evident at 140 bp, whereas the mutant gene was observed at 280 bp. (B) PCR analysis of the *Rag1* gene. The PCR product of the WT gene was evident at 474 bp, whereas that of the mutant gene was observed at 530 bp. **Figure S2. Endogenous islet function was unaffected by insulin treatment.** Insulin-treated AKITA mice 1 week after the removal of the insulin implant underwent the IPGTT (A, B), GSIS analysis (C), and ITT (D) to test their glucose and insulin tolerance in comparison to the untreated AKITA mice and WT controls, respectively. (A, B) After discontinuing insulin treatment, AKITA mice were glucose intolerant, similar to the untreated mice (A), also revealed by the AUC shown in (B). (C) The insulin implants contained insulin from different species, which induces anti-insulin antibodies, thus interfering with insulin measurements. Therefore, immunodeficient *Rag1*−/− Akita mice were used to avoid interference. No increase in endogenous blood insulin levels were detected in the Akita mice after insulin treatment was discontinued. (D) The relative rates of glucose decrease as a result of insulin treatment from each basic glucose level. n. s., not significant; **p<0.01, two-tailed unpaired Students’ *t*-test.
(PDF)
