# Supplementary material for: Beneficial Effect of Insulin Treatment on Islet Transplantation Outcomes in Akita Mice
Source: PLoS One. 2014 Apr 17;9(4):e95451. doi: 10.1371/journal.pone.0095451 (PMC3990632; doi:10.1371/journal.pone.0095451)
Supplement: File S1 — includes the following: Figure S1. Genotyping of Akita mice. (A) PCR analysis of the Ins 2 gene. The PCR product of the WT gene was evident at 140 bp, whereas the mutant gene was observed at 280 bp. (B) PCR analysis of the Rag1 gene. The PCR product of the WT gene was evident at 474 bp, whereas that of the mutant gene was observed at 530 bp. Figure S2. Endogenous islet function was unaffected by insulin treatment. Insulin-treated AKITA mice 1 week after the removal of the insulin implant underwent the IPGTT (A, B), GSIS analysis (C), and ITT (D) to test their glucose and insulin tolerance in comparison to the untreated AKITA mice and WT controls, respectively. (A, B) After discontinuing insulin treatment, AKITA mice were glucose intolerant, similar to the untreated mice (A), also revealed by the AUC shown in (B). (C) The insulin implants contained insulin from different species, which induces anti-insulin antibodies, thus interfering with insulin measurements. Therefore, immunodeficient Rag1−/− Akita mice were used to avoid interference. No increase in endogenous blood insulin levels were detected in the Akita mice after insulin treatment was discontinued. (D) The relative rates of glucose decrease as a result of insulin treatment from each basic glucose level. n. s., not significant; **p<0.01, two-tailed unpaired Students’ t-test. (PDF) [file pone.0095451.s001.pdf]

Figure S1

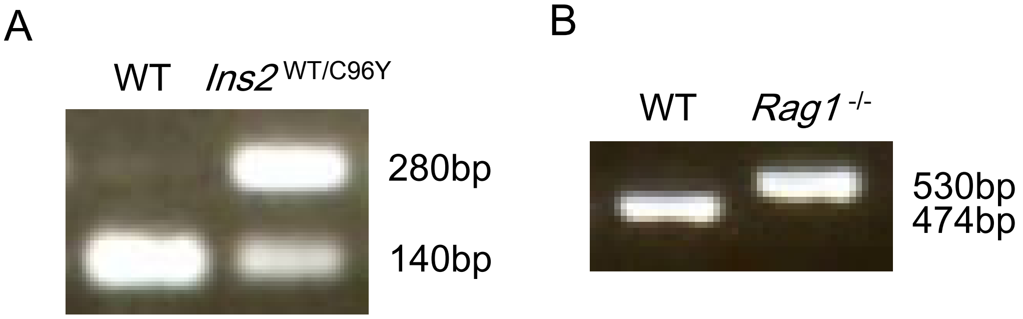

Figure S2

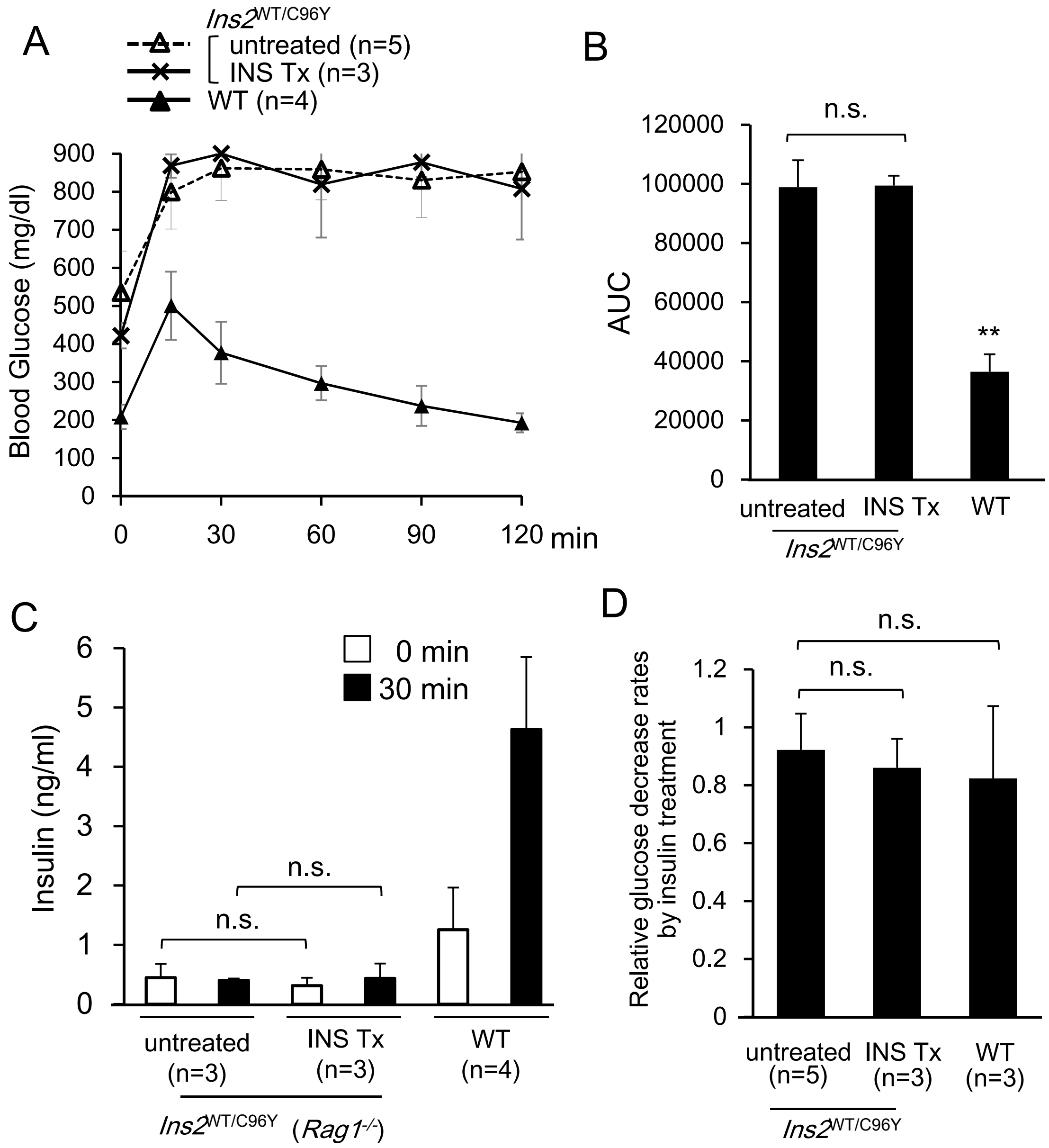
A
Ins2WT/C96Y
untreated (n=5)
INS Tx (n=3)
WT (n=4)
Blood Glucose (mg/dl)
900
800
700
600
500
400
300
200
100
0
0
30
60
90
120 min
B
n.s.
120000
100000
80000
60000
40000
20000
0
AUC
**
untreated
INS Tx
WT
Ins2WT/C96Y
C
Insulin (ng/ml)
6
5
4
3
2
1
0
0 min
30 min
n.s.
n.s.
untreated (n=3)
INS Tx (n=3)
WT (n=4)
Ins2WT/C96Y (Rag1-/-)
D
n.s.
n.s.
Relative glucose decrease rates by insulin treatment
1.2
1
0.8
0.6
0.4
0.2
0
untreated (n=5)
INS Tx (n=3)
WT (n=3)
Ins2WT/C96Y

Supporting Information Legends

Figure S1. Genotyping of Akita mice

(A) PCR analysis of the *Ins 2* gene. The PCR product of the WT gene was evident at 140 bp, whereas the mutant gene was observed at 280 bp. (B) PCR analysis of the *Rag1* gene. The PCR product of the WT gene was evident at 474 bp, whereas that of the mutant gene was observed at 530 bp.

Figure S2. Endogenous islet function was unaffected by insulin treatment

Insulin-treated AKITA mice 1 week after the removal of the insulin implant underwent the IPGTT (A, B), GSIS analysis (C), and ITT (D) to test their glucose and insulin tolerance in comparison to the untreated AKITA mice and WT controls, respectively. (A, B) After discontinuing insulin treatment, AKITA mice were glucose intolerant, similar to the untreated mice (A), also revealed by the AUC shown in (B). (C) The insulin implants contained insulin from different species, which induces anti-insulin antibodies, thus interfering with insulin measurements. Therefore, immunodeficient *Rag1*$^{-/-}$ Akita mice were used to avoid interference. No increase in endogenous blood insulin levels were detected in the Akita mice after insulin treatment was discontinued. (D) The relative rates of glucose decrease as a result of insulin treatment from each basic glucose level. n. s., not significant; $^{**}p<0.01$, two-tailed unpaired Students' *t*-test.
